# Study protocol for a randomized controlled trial of the RASPERA project: recalling and anticipating specific positive events to boost resilience in adolescents

**DOI:** 10.3389/fpubh.2023.1216988

**Published:** 2023-11-24

**Authors:** Eline Loyen, Liesbeth Bogaert, David John Hallford, Arnaud D'Argembeau, Filip Raes

**Affiliations:** ^1^Faculty of Psychology and Educational Sciences, University of Leuven, Leuven, Belgium; ^2^KU Leuven Child and Youth Institute, KU Leuven, Leuven, Belgium; ^3^School of Psychology, Deakin University, Melbourne, VIC, Australia; ^4^Psychology and Neuroscience of Cognition Research Unit, Department of Psychology, University of Liège, Liège, Belgium

**Keywords:** adolescence, resilience, mental wellbeing, specific memories, specific future events, positive affect, positive affect regulation, anhedonia

## Abstract

**Background:**

Many stress-related mental health problems, like depression and anxiety, emerge during adolescence, with some recent studies suggesting numbers are increasing. One possible way to reduce adolescents' vulnerability to stress-related mental health problems is to increase their resilience by training them in recalling specific positive memories and anticipating specific positive future events. Therefore, an innovative *combi*-training (called Positive Events Training; PET) was developed, focusing on the enhancement of the specificity of *both* past and future positive autobiographical events in adolescents. Its effects on adolescents' resilience and mental wellbeing will be examined.

**Methods:**

A cluster randomized controlled trial with a 2 (condition) × 3 (time-point) factorial design was conducted. Classes of adolescents were randomized to either a PET program (intervention) or a CREAtive writing Training (CREAT) program (active control). Both trainings consisted of four sessions of 50 min (one session, weekly, for four consecutive weeks) and were delivered in schools. Before (pre-training, T1), immediately after (post-training, T2), and 2 months after the training (follow-up, T3), participants completed a series of self-report questionnaires. Primary outcomes are resilience and mental wellbeing. Secondary outcomes are positive affect, positive affect regulation and anhedonia. CONSORT criteria for conducting and reporting RCTs will be used.

**Ethics and dissemination:**

Ethical approval was obtained from the Social and Societal Ethics Committee (SMEC) and the study has been preregistered on Open Science Framework (OSF) and ClinicalTrials.gov (Trial registration number: NCT05757180). We plan to develop a free, online, web-based self-directed PET protocol for teachers if the study provides evidence for the effectiveness of the PET program in increasing adolescents' resilience and mental wellbeing, so teachers can deliver the program to future students without the need of professional external trainers.

## 1 Introduction

### 1.1 Rationale

Many stress-related mental health problems emerge during adolescence and represent the largest cause of the burden of disease among young people (1). Furthermore, there has been a significant increase in the prevalence of anxiety between 2012 and 2018 (2) and of depression symptoms over the past 20 years (3) in adolescents. Such problems have a profound and often lasting impact on young people's future (1).

One important way to reduce the burden of disease due to stress-related mental disorders is to shift the focus from addressing deficits to strengthening protective factors. This fits with the call to complement a treatment approach with a preventative approach (4). Our study adopts such a preventative approach, examining ways to strengthen adaptive and protective factors. An important protective factor in this context is resilience, which is the capacity to bounce back from stressful life events. It is associated with better mental wellbeing (5), and with higher quality of life in adolescents (6). Strengthening factors related to resilience can, thus, be expected to improve mental wellbeing.

Recent basic research indicates that recalling specific positive memories is one such resilience-enhancing factor, both in adults and adolescents. Studies show that recalling specific positive memories is associated with reduced vulnerability to emotional problems in the face of stress (7), actively buffers the physiological and emotional consequences of stress (8), repairs negative mood (9, 10), and is associated with improvement in problem solving and hopelessness (11). The recall of specific positive memories attenuates negative self-cognitions and dampens cortisol responses after exposure to stress (7, 8). These findings align with pilot studies showing that recalling specific positive memories has positive effects on resilience and mental wellbeing. For example, positive MEmory Specificity Training (MEST) has been found to facilitate letting go of negative self-thoughts in adolescents compared to a control training (12).

In a similar vein, research suggests that anticipating specific future positive events holds promise as a potential focus for resilience and mental wellbeing promoting interventions. Basic research in young adults shows, for example, that anticipating positive events leads to improved coping and recovery following stress (13). Such findings align with results from an initial intervention study and clinical trial in depression showing that Future Event Specificity Training (FEST) in adults leads to increased anticipated and anticipatory pleasure for future events and a stronger perception that one has control over future events, which are essential ingredients to mitigate the negative influence of stressful events (14, 15).

So far, studies have focused either on increasing specificity of memories *or* future events, while it has become clear that both have unique, yet complimentary benefits. Whereas (positive) memory specificity has been linked to less negative thinking (7, 12), improved problem-solving (16, 17), positive self-esteem (18) and reduced depressive symptomatology (19), positive future specificity has been linked to less impulsive behavior in adolescents (20, 21) and emotional states that motivate us to engage in rewarding, more meaningful behaviors that maintain good mental health (15, 22–24). Therefore, we will test for the first time the effects of an innovative *combi*-training (called Positive Events Training; PET), focusing on the enhancement of the specificity of *both* past and future positive events in adolescents. Studies showed that there is considerable overlap in the brain structures that are involved in thinking of past and future events. A recent meta-analysis showed that both processes of past and future thinking have cerebral bases that involve the medial temporal lobe (including the hippocampus), as well as frontal, temporal, and parietal areas (25). Interestingly, however, some of these regions were consistently more strongly engaged during future thinking than remembering, suggesting that the former involves additional constructive processes (26). The added value of working with memories and future events is that it will allow people to integrate past, present, and future experiences, which is important for creating a sense of meaning, purpose, and direction in one's life, which contributes to mental wellbeing.

There appear to be many candidate mechanisms by which the representation of specific positive events is believed to promote resilience and wellbeing (7, 27). However, one key mechanism, both for past and future positive events, is thought to be positive emotions. Research shows that the ability to increase positive emotions plays an essential role in resilience (8, 13). One of the key reasons why resilient individuals are able to bounce back more effectively from stressful experiences is that they use positive emotions when dealing with stress (28). Positive emotions buffer against stress and strengthen people's coping repertoire (29). Importantly, such emotions can be induced via the recollection and anticipation of positive events (8, 13, 14, 23).

### 1.2 Hypotheses

The first and main aim is to examine the effects of the PET program on resilience and mental wellbeing as primary outcomes. It is expected that PET will cause an increase in resilience and mental wellbeing at post-intervention and at follow-up. The second aim is to examine the effects of the PET program on secondary outcomes related to positive emotions, as positive emotions are believed to be a central way via which the PET program leads to increased resilience and better mental wellbeing. In particular, we focus on (i) the present moment experience of positive emotions, (ii) the regulation of positive emotions when they are experienced, and (iii) the basic capacity to experience pleasure (or the lack of that capacity, called anhedonia).

So besides looking at the experience of positive emotions as such, we will also explore the impact of the PET program on how adolescents regulate such positive emotions. This is an important focus, as research shows that regulation of positive emotions is also a prominent factor in the development or prevention of emotional problems. For example, youngsters who typically dampen or downplay positive emotions (by using thoughts such as “These good feelings won't last”) are more likely to suffer mental ill-health (30, 31). On the contrary, youngsters who deploy more of a savoring response (e.g., “I'm attempting to fully feel and enjoy this positive emotion”) are typically better off when it comes to mental wellbeing, as savoring responses amplify and extend positive emotions (30, 31).

In the context of positive emotion and its regulation, we will also examine the impact of the PET program on anhedonia. In contrast to dampening or savoring, anhedonia refers to a lack of capacity to experience interest or pleasure in activities that might otherwise be thought to be rewarding (32). In addition, it refers to the diminished pleasure from anticipation to future positive events and to the decreased motivation to pursue positive outcomes. The reduced perception of reward or pleasure in young people has been linked to diverse negative outcomes, reflecting varying degrees of mental ill-health (33–36). Fortunately, the ability to imagine positive future events, which is targeted in the PET program, is associated with both the anticipation and present-moment experience of pleasure (37). So, examination of the effects of the PET program on anhedonia will help to establish whether the training affects not only the experience of positive emotion and regulatory processes, but also the underlying capacity to experience them. Furthermore, according to Hallford et al. (15), reducing anhedonia can promote mental wellbeing. Future Event Specificity Training (FEST) seems to be an intervention that can lead to less anhedonia and an increase in anticipatory and anticipated pleasure. At 3 months follow-up, other clinical and mental wellbeing outcomes were also found (e.g., less likelihood of reaching threshold for a depressive episode, increased behavioral activation, improved global functioning) (15).

## 2 Materials and methods

### 2.1 Study design and sample size

A cluster randomized controlled trial with a 2 (condition; PET program vs. CREAT program) × 3 (time point; baseline, post-training, and follow-up) factorial design was conducted. Classes of adolescents were asked to complete measures of the study outcomes at all three time points (see procedure).

A total sample of 130 adolescents (i.e., *N* = 65 for each condition) provides 80% statistical power to investigate the effectiveness of the PET program, with an alpha of 0.05 and estimated medium effect sizes of 0.25 (38). Given previous (large) effect sizes ranging from *d* = 0.82 (for increased specificity) to *d* = 1.78 (for anticipated pleasure) (11, 14), this more conservative effect size estimate will help mitigate against Type II errors. However, in anticipation of considerable drop-out rates, some oversampling was done. We intended to recruit 15% extra participants (based on drop out of ±17% in previous school study of our lab), resulting in an sample size of approximately 150 participants (75 per group).

### 2.2 Participants

Participants were recruited via secondary schools in Flanders, Belgium. Years 1 to 4 (1st and 2nd grade) of general secondary education were invited to participate, referring to ages 12 – 16. Five different schools (spread across three provinces) participated in the study. Each school selected one pair of parallel classes, which resulted in five pairs of classes (two of the first year, one of the second year, and two of the third year). In total, 188 adolescents participated in the study. Of all the adolescents, 95 were in the PET program (*M*_age_ = 13.2, SD_age_ = 1.00) and 93 in the CREAT program (*M*_age_ = 13.3, SD_age_ = 1.08). Gender distribution was approximately the same in both groups (PET program: 73.6% females, 25.3% males, 2.1% other; CREAT program: 76.3% females, 22.6% males, 1.1% other).

### 2.3 Procedure

The procedure of the RASPERA-study is shown in the SPIRIT schedule of enrolment, interventions, and assessments (Supplementary Figure 1). Several secondary schools were invited for study participation via email. Schools that expressed an interest in the study received more detailed information, in-person at school or via an online meeting. Following the selection of parallel classes by the schools, one of the involved researchers gave a short oral presentation at the schools to inform the adolescents about the background of the study, procedure, potential risks and benefits, the voluntary character of participation and information about the informed consent procedure. In this way, adolescents were informed about all study phases and potential consequences, and they had the opportunity to directly ask for clarification if needed. In case any questions arise afterwards, contact information of the researchers was shared.

Immediately following the oral presentation, each adolescent received two printed booklets with a summary of the study and the informed consent form in duplicate (i.e., exemplar researcher and adolescent/parent). One booklet was addressed to the adolescent and the other to their parent(s). Adolescents and their parents were invited to sign their versions of the informed consent in duplicate. In consultation with the school, a deadline was agreed on for the adolescents to hand in the two signed versions of the informed consent. Direct access to the school communication platform was asked for as contact information of participants and their parents is integrated in this platform, which was needed to provide participants with their unique participation code.

Subsequently, participants completed the baseline assessment online, which consisted of a series of self-report questionnaires. It is only after completion of this first assessment that the researcher who was coordinating the study in the schools and who delivered the trainings was informed about the result of the random allocation (conducted by an independent statistician) of classes to one of the two conditions. The training programs started 1 week after the first assessment, and, in every school, classes received training sessions on the same days. Furthermore, training sessions were delivered by a researcher without a teacher being present. In that way, contextual variation across sessions and classes was kept to a minimum (e.g., no confounding effects related to the presence of a particular teacher). In addition, efforts were made to ensure that both trainings closely resembled in terms of format, structure, intensity, interaction between adolescents, invitations to practice with the skills taught during the in-class sessions. In this way, we aimed to prevent that adolescents would start to speculate about study hypotheses.

After completion of the trainings, adolescents of both groups took part in two final assessments. That is, a similar series of self-report questionnaires were presented 1 week (post-training) and, again, 2 months after the final training session (follow-up). Assessments in parallel classes (i.e., same school) were administered at the same day to minimize potential confounding effects by contextual factors. Per assessment, completing the self-report questionnaires took approximately 60–90 min. Self-report questionnaires were administered via Qualtrics (GDPR-proof online survey platform) on the laptop/tablet of the participants at school, with the intention to minimize errors due to manually entering data and missing values (i.e., “request response” option if questions were not completed). One of the involved researchers was present during the assessments, so participants could ask questions if necessary.

At the end of the study, participants will receive a €25-voucher for Bol.com (a local online store) as an incentive. In addition, participants and participating schools will be debriefed via email about our specific hypotheses and they will have the possibility to contact the researchers for additional information. Participating schools will automatically be informed about the general results.

### 2.4 Training programs

#### 2.4.1 Positive Events Training (PET)

The PET program is a group-based training program combining Memory Specificity Training [MEST; (39)] and Future Event Specificity Training [FEST; (14)]. It comprises four sessions of 50 min that are delivered for four consecutive weeks. The training is delivered in a standardized manner, using the PET program manual developed for this study, with content adapted from our MEST and FEST manuals. Following brief psychoeducation on the rationale behind the PET program (i.e., recalling and anticipating specific positive events has been found to be beneficial for mental wellbeing), participants practice in generating detailed specific memories (Sessions 1 and 2) and future events (Sessions 3 and 4) using neutral and positive cue-words. Homework, including exercises that resemble those used in the training sessions, is provided after every session to stimulate adolescents to further practice at home. During the sessions the trainer explained the homework exercises to the adolescents and encouraged the adolescents to actively engage with the homework material by emphasizing its importance. At the start of each session, the trainer checked if everyone completed their homework which meant that adolescents also were aware that completion of homework was being monitored. Moreover, adolescents received additional practice material after the final training session to continue practicing their skills.

#### 2.4.2 CREAtive writing Training (CREAT)

The control training or CREAtive writing training (CREAT) follows the exact same format and length as the PET program (i.e., delivered by a trainer in group over 4 × 50-min sessions, delivered for four consecutive weeks, including homework exercises after each session and additional material at the end of the training). Following brief psychoeducation on the rationale behind the PET program, participants completed a series of creative writing exercises – which are not self-referential – using funny and thought-provoking writing prompts. They were explicitly instructed not to write about themselves as the main character in their stories, to mitigate against the risk of partially triggering expected working mechanisms of the PET program. Concerning the rationale, participants were told that creative writing has been found to be beneficial for mental wellbeing, as they cultivate creativity and stimulate imagination skills. A similar CREAT program has been successfully used before in an online version as a control training for a memory specificity training (12).

### 2.5 Outcomes

Primary outcomes are resilience and mental wellbeing. Secondary outcomes are positive affect, positive affect regulation and anhedonia.

#### 2.5.1 Self-report questionnaires

In the self-report questionnaires below adolescents were instructed to consider and rate their experiences of the past 2 weeks. All scales have proven psychometric quality (see below) and have been used successfully in adolescents before (6, 40–43). Means of the scales will be calculated as final scores of the questionnaires.

The short version of the Connor-Davidson Resilience Scale [CD-RISC (44–46) translated into Dutch by Danhof-Pont and Schrier (47)] was used to assess resilience (primary outcome). This scale consists of 10 items (e.g., “Dealing with stress makes me stronger”) that are rated on a 5-point scale going from 1 (*not at all*) to 5 (*very often*), with higher scores indicating higher resilience (total score: minimum = 10, maximum = 50). The CD-RISC shows good construct validity, convergent and divergent validity, test-retest reliability, and good internal consistency with a Cronbach's alpha value of 0.85 in young adults (44, 45).

Mental wellbeing (primary outcome) was measured using the Short Warwick-Edinburgh Mental Wellbeing Scale (SWEMWBS) (48, 49). The SWEMWBS consists of seven statements (e.g., “I was optimistic about the future.” or “I felt useful.”) that are scored on a 5-point scale ranging from 1 (*never*) to 5 (*always*). Higher scores reflect higher mental wellbeing (total score: minimum = 7, maximum = 35). There is evidence for good convergent, construct and discriminant validity and for high internal consistency (α = 0.88) in an adolescent sample (48, 50, 51).

The Positive Affect subscale of the Positive and Negative Affect Schedule Scales (PANAS) was administered to assess positive affect (secondary outcome) (52, 53). The Positive Affect subscale of the PANAS consists of 10 items (words) that describe positive feelings (e.g., “excited”). Items are rated on a 5-point scale going from 1 (*very slightly or not at all*) to 5 (*extremely*), with higher scores reflecting higher positive affect (total score: minimum = 10, maximum = 50). The scale has good convergent and discriminant validity and good internal consistency (Cronbach's alpha of 0.79) in a sample of adolescents (52–54).

The Responses to Positive Affect scale, child version (RPA-C) (55–57) was used to assess positive affect regulation (secondary outcome). The RPA-C consists of items reflecting both dampening (e.g., “When you felt happy, how often did you think: ‘I don't deserve this'?”) and savoring responses (e.g., “When you felt happy, how often did you notice that you felt full of energy?”). All 17 items are rated on a 4-point scale ranging from 1 (*not at all*) to 4 (*very often*). The minimum total score a participant can get on the RPA-C is 17, the maximum is 68. There is evidence for good convergent and incremental validity and internal consistency for each scale is sufficient to good (Cronbach's alpha ranges from 0.72 to 0.86) in both children and (young) adults (55–57).

Symptoms of anhedonia (secondary outcome) were measured using the Leuven Anhedonia Self-report Scale (LASS, 2nd version) (58). All 12 items (e.g., “There were few things I looked forward to.”) are rated on a 5-point scale going from 1 (*completely untrue*) to 5 (*completely true*) with higher scores reflecting higher levels of anhedonia (total score: minimum = 12, maximum = 60). The total scale of the LASS has good internal consistency with a Cronbach's alpha value of 0.81 (58) and there is evidence for good convergent and discriminant validity in a sample of adolescents (31).

The Depression Anxiety Stress Scale (DASS-21) (59) was used to assess basic mental health to control for baseline differences in the training groups, and to explore the generalized effect of the PET program on levels of depressive symptoms and stress at post-training and at follow-up. The DASS-21 consists of 21 items (e.g., “I felt like my life had no meaning.”) that are rated on a 4-point scale going from 0 (*never*) to 3 (*almost always*), with 0 as a minimum total score, and 63 as a maximum total score. There is evidence for good construct and concurrent validity and good internal consistency of the subscales of the DASS-21 in both adolescent and adult samples, with Cronbach's alpha ranging from 0.79 to 0.94 (60, 61).

#### 2.5.2 Manipulation checks

The Episodic Future Thinking Test (EFT-T) (14, 62) was used to assess episodic future thinking specificity. For each assessment point, a different set of eight cue positive or neutrally-valenced words (e.g., love, street) were presented. The cue-word sets were composed of cue words balanced in their valence, arousal and frequency of occurrence based on Moors et al.'s (63) norms for Dutch words. Alternately, participants were asked to generate a positive future event that is related to a positive or neutral cue word (i.e., positive – neutral – positive – neutral cue word etc.). For this study, the original EFT-test was slightly adapted as participants were asked to generate *only* potential positive future events regardless of the cue word valence.

The Autobiographical Memory Test (AMT) (39) measured positive memory specificity. Similar to the EFT-T, the AMT was slightly adapted compared to the original version: participants were asked to write down one specific positive memory the cue-word reminds them of regardless of the cue-word valence (parallel to the EFT, four positive and four neutral in an alternating order). For the AMT, the same sets of cue words as in the EFT-test were presented to participants. However, cue words used in the EFT-test and AMT of the same assessment point were different. Also, the cue-word set used in the AMT at T1 was not used for the EFT-test at T2, as retrieval of positive memories at T1 might have influenced the process of generation of positive future events at T2 (e.g., rewording memories into positive future events instead of simulating new expected positive events).

#### 2.5.3 Non-validated questionnaires

The AMT and EFT-test were complemented by a series of single-item ratings (14, 39) following each cue-word. First, two single-item ratings of the anticipated/anticipatory (future) or remembered/felt pleasure (past) associated with the events participants recall/generate on the AMT and EFT-T were administered. Second, participants were asked to complete two single-item ratings of the level of detail and mental imagery associated with the events they recall/generate on the AMT and EFT-T. As part of the EFT-test only, two additional single-item ratings of perceived control and perceived likelihood of occurrence associated with the generated future events were assessed. Finally, two additional single-item ratings of anticipated/anticipatory (future) or remembered/felt positive feelings (i.e., not solely pleasure) (past) associated with the events participants recall/generate on the AMT and EFT-T were administered. Mean scores of these single-item ratings will be calculated.

The Leuven Exeter Dampening Scale-General (LEDS-G; Bogaert et al., unpublished) is a newly developed self-report scale, aimed at covering a larger variety of dampening appraisal styles compared to traditionally used scales. The LEDS-G consists of 13 items (e.g., “I can only be happy if others are too.”) that are rated on a 5-point scale going from 1 (*almost never*) to 5 (*almost always*), with 13 as a minimum total score, and 65 as a maximum total score.

A Dutch (non-validated) version of the 10-item-version of Abridged Ways of Savoring Checklist for Adolescents (savoring scales) (64) was used to assess savoring. Items (e.g., “I looked for other people to share it with.”) are rated on a 7-point scale going from 1 (*totally disagree*) to 7 (*totally agree*), with higher scores reflecting higher levels of savoring (total score: minimum = 10, maximum = 70).

As part of the second assessment point, adolescents were asked about the extent to which they actively engaged in the training and actually completed the homework exercises. Additionally, some questions about their personal experiences with the training were presented. As part of the final assessment, adolescents were asked to which extent and how they continued practicing the skills they learned during the training. Finally, they were asked a few questions about the perceived impact of the training.

### 2.6 Harms

The EFT-test and AMT are not expected to have possible disadvantages for participants, as they do not directly assess (expressions of) mental health or wellbeing. Instead, they explicitly focus on positive generated future thoughts and memories. Some of the self-report questionnaires do tap into aspects of mental health (e.g., resilience, mental wellbeing, anhedonia) though, which may result in some adolescents becoming aware of their engagement in suboptimal ways of regulating positive emotions, low levels of positive emotions, etc. However, such gained insights in one's own mental wellbeing and ways of regulating emotions should not necessarily be considered problematic. It may rather encourage participants to pay (more) attention to their mental wellbeing and ways to enhance it.

Irrespective of the experienced impact – if any – of the baseline assessment, the intervention group was offered the PET program immediately afterwards. The PET program aims to stimulate resilience and mental wellbeing, increase levels of positive emotions, and create a shift toward more adaptive ways of positive emotion regulation. In addition to participants of the intervention group, participants of the control group may benefit from their active control training (CREAT). We do not rule out the possibility that participants in the CREAT program would also experience (some) positive effects from that training by focusing on positive emotions (since they are asked to write down funny and non-realistic stories) and non-training-related factors of the training. Furthermore, as part of the informed consent, participants were provided with a list of relevant contact points if they experience the need for counseling (including specific services for youngsters such as www.awel.be, tel. 102; www.tele-onthaal.be; jongerenadviescentra). This information was referred to again at the three assessment phases.

Concerning the trainings, both the PET and CREAT programs were provided by one of the involved researchers (EL), who has a background in clinical psychology (master degree). Additionally, regular supervision was organized by FR (professor, clinical psychologist and behavioral therapist) and the back-up trainer (LB), both closely involved in the developmental process of the PET and CREAT programs. Ultimately, no emerging safety issues or adverse events occurred. Nevertheless, if this had been the case, this would have been discussed immediately with the PI (FR) and documented via the available adverse event form for emerging safety issues.

However, no inconveniences are expected to be caused by the PET program. Previous research, cited above, has already demonstrated the beneficial impact of both components (MEST and FEST) on several outcomes (e.g., depressive symptoms, anhedonia), and the PET program is explicitly focused on the elaboration of positive personal experiences. As mentioned above, also for the CREAT program no indications for negative consequences were found in a previous study (12). Finally, a remuneration tries to compensate for possible, though unlikely, inconveniences.

### 2.7 Data management

Age and demographic data (month of birth, year of birth, gender, ethnicity, school, class, grade) were recorded. Participants were asked to fill in a unique participant code (received via the school communication platform before the actual assessment), so their name cannot be directly linked to the data collected in this study (i.e., in the same Qualtrics survey). As soon as data collection is finished (including linking of data of the three assessment points), the file in which names of participants are linked with the unique codes, will be deleted.

The school communication platform was used to send participants their unique codes and practical information about the study. As part of both trainings, participants were handed over a printed version of the exercise sheets (during sessions) and worksheets (homework). If needed, a digital version of the worksheets was sent via email. All contact information will be deleted as soon as data collection is finished. As part of the informed consent, participants could fill in their email address if they want to be informed about the general study findings. After communication of the findings, this list with contact information will be deleted.

Qualtrics, the platform that was used for the assessments, is GDPR compliant and all surveys were created on a KU Leuven licensed (protected) Qualtrics-account. As data of a personal and sensitive nature require to be transferred, stored, and handled with optimal guarantee for security and in accordance with EU privacy regulations, the data from the self-report questionnaires will be analyzed based on unique participant codes.

### 2.8 Statistical analysis plan

All analyses will be completed in accordance with CONSORT standards. Intent-to-treat analysis will be used. Our primary and secondary outcomes will be analyzed using analysis of variance (mixed ANOVA) with time (pre, post, 2-month follow-up) as a within-subjects factor, and condition (PET, CREAT) as a between-subjects factor. Any baseline differences will be covaried in analyses.

To take into account the repeated measure nature of the study, group differences for the continuous variables at post-training and at follow-up will be tested via a mixed ANOVA model. A model with a main effect for time and for group (PET program vs. CREAT program), random effects for the dummy variables representing time clustered within individuals, and an interaction between time and group will be run to detect changes between groups for the primary and secondary outcomes. Follow-up independent samples *t-*tests will be calculated to examine group differences at post-training and follow-up time points. To correct for the inflated risk of a Type I error from multiple comparisons, Benjamini and Hochberg's (65) false discovery rate procedure will be applied to the main outcomes (i.e., primary and secondary outcomes). Although sample size planning was based on mixed ANOVA modeling, multilevel analyses that take into account the dependence in the data at school-level (clusters) will also be conducted to account for possible variance due to school membership.

### 2.9 Ethical considerations and declarations

Ethical approval for this study was obtained from the Social and Societal Ethics Committee (SMEC; https://admin.kuleuven.be/raden/en/smec). Subsequent modifications to the protocol were submitted to SMEC. Moreover, this study has been preregistered on Open Science Framework (OSF; https://osf.io/6acy2) and ClinicalTrials.gov (NCT05757180).

### 2.10 Status and timeline

The data collection has now been completed. The first assessment (baseline) took place in January 2023, the second assessment (post-training) in March 2023, and the third and last assessment (follow-up) in May 2023 (Table 1). The coded, pseudonymized dataset will be uploaded in a csv format to Open Science Framework (in a restricted access repository) upon publication of the research results.

**Table 1:** Study timeline of the RASPERA-study.

**Approval of Social and Societal Ethics Committee (SMEC KU Leuven)**	**First approved on June 27, 2022 (a number of amendments were approved in the period between July 2022 and January 2023)**
Preregistration on Open Science Framework (OSF)	September 27, 2022
Recruitment of schools/participants	September–December 2022
Enrollment of first school/participants	October 21, 2022
Short oral presentation at the schools	January 9–13, 2023
Baseline assessment	January 23–27, 2023
First training session	January 31–February 3, 2023
Second training session	February 6–10, 2023
Third training session	February 13–17, 2023
Preregistration on ClinicalTrials.org	February 4, 2023
Fourth and last training session	February 27–March 3, 2023
Post-training assessment	March 6–10, 2023
Follow-up assessment	May 8–12, 2023

## 3 Discussion

### 3.1 Strengths and limitations

The RASPERA-study uses a cluster randomized controlled trial design to investigate the effect of the PET program on adolescents' resilience and their mental wellbeing as primary outcomes, compared to a control training. Furthermore, the RASPERA-study examines potential underlying mechanisms of the impact of the PET program on resilience and mental wellbeing, with a particular focus on positive emotions and related emotion regulation styles. In addition, the RASPERA-study is the first study to investigate the effect of a *combi*-training (PET program) that focuses on the enhancement of the specificity of both past and future positive events in adolescents. Working with both past and future positive events is expected to allow people to integrate past, present, and future experiences. This is important for creating a sense of meaning, purpose, and direction in one's life. Finally, in addition to the post-training assessment, the RASPERA-study includes a follow-up assessment to examine the impact of the PET program on the study outcomes 2 months after the training.

One limitation of the study design is that, due to the primary focus on resilience and mental wellbeing, data collection solely relies on self-report measures. Furthermore, generalizability of the results might be somewhat limited, because for this initial examination of the effectiveness of the PET program only secondary schools in Flanders (Belgium) participated in the study. However, this initial test is crucial to find support for the effectiveness of the PET program on the study outcomes. In a next step, future studies should further investigate the applicability of the PET program in other contexts.

### 3.2 Dissemination plan

We plan to share consolidated findings in an article, which will be submitted for publication in a peer-reviewed journal. Furthermore, we plan to develop a free, online, web-based self-directed PET protocol for teachers if the study provides evidence that the PET program is effective in increasing adolescents' resilience and mental wellbeing, so teachers can deliver the program to future students without the need of professional external trainers.

## Ethics statement

The studies involving humans were approved by the Social and Societal Ethics Committee (SMEC). The studies were conducted in accordance with the local legislation and institutional requirements. Written informed consent for participation in this study was provided by the participants' legal guardians/next of kin.

## Author contributions

LB, FR, DH, and AD were involved in conception and study design. EL and LB were responsible for drafting the article and collected the data and they will analyze them. EL was responsible for recruitment of the participants. All authors were involved in critical revision of the article, approved the final version of the article, and agreed to be accountable for all aspects of the work.

## Supplementary material

Supplementary Figure 1 SPIRIT schedule of enrollment, interventions, and assessments.
