# Corrigendum: Study protocol for a randomized controlled trial of the RASPERA project: recalling and anticipating specific positive events to boost resilience in adolescents

**DOI:** 10.3389/fpubh.2024.1360159

**Published:** 2024-01-29

**Authors:** Eline Loyen, Liesbeth Bogaert, David John Hallford, Arnaud D'Argembeau, Filip Raes

**Affiliations:** ^1^Faculty of Psychology and Educational Sciences, University of Leuven, Leuven, Belgium; ^2^KU Leuven Child and Youth Institute, KU Leuven, Leuven, Belgium; ^3^School of Psychology, Deakin University, Melbourne, VIC, Australia; ^4^Psychology and Neuroscience of Cognition Research Unit, Department of Psychology, University of Liège, Liège, Belgium

**Keywords:** adolescence, resilience, mental wellbeing, specific memories, specific future events, positive affect, positive affect regulation, anhedonia

In the published article, there was an error in the Funding statement. The funding number for the Research Foundation – Flanders (FWO-Vlaanderen) under a Red Noses grant incorrectly appeared as “G049019N”. The corrected Funding statement appears below.

## Funding

This research was supported by the Research Foundation – Flanders (FWO-Vlaanderen) under a Red Noses grant (G0D5522N).

The authors apologize for this error and state that this does not change the scientific conclusions of the article in any way. The original article has been updated.

