# Supplementary figures and images for: Study protocol for a randomized controlled trial of the RASPERA project: recalling and anticipating specific positive events to boost resilience in adolescents
Source: Front Public Health. 2023 Nov 24;11:1216988. doi: 10.3389/fpubh.2023.1216988 (PMC10704171; doi:10.3389/fpubh.2023.1216988)

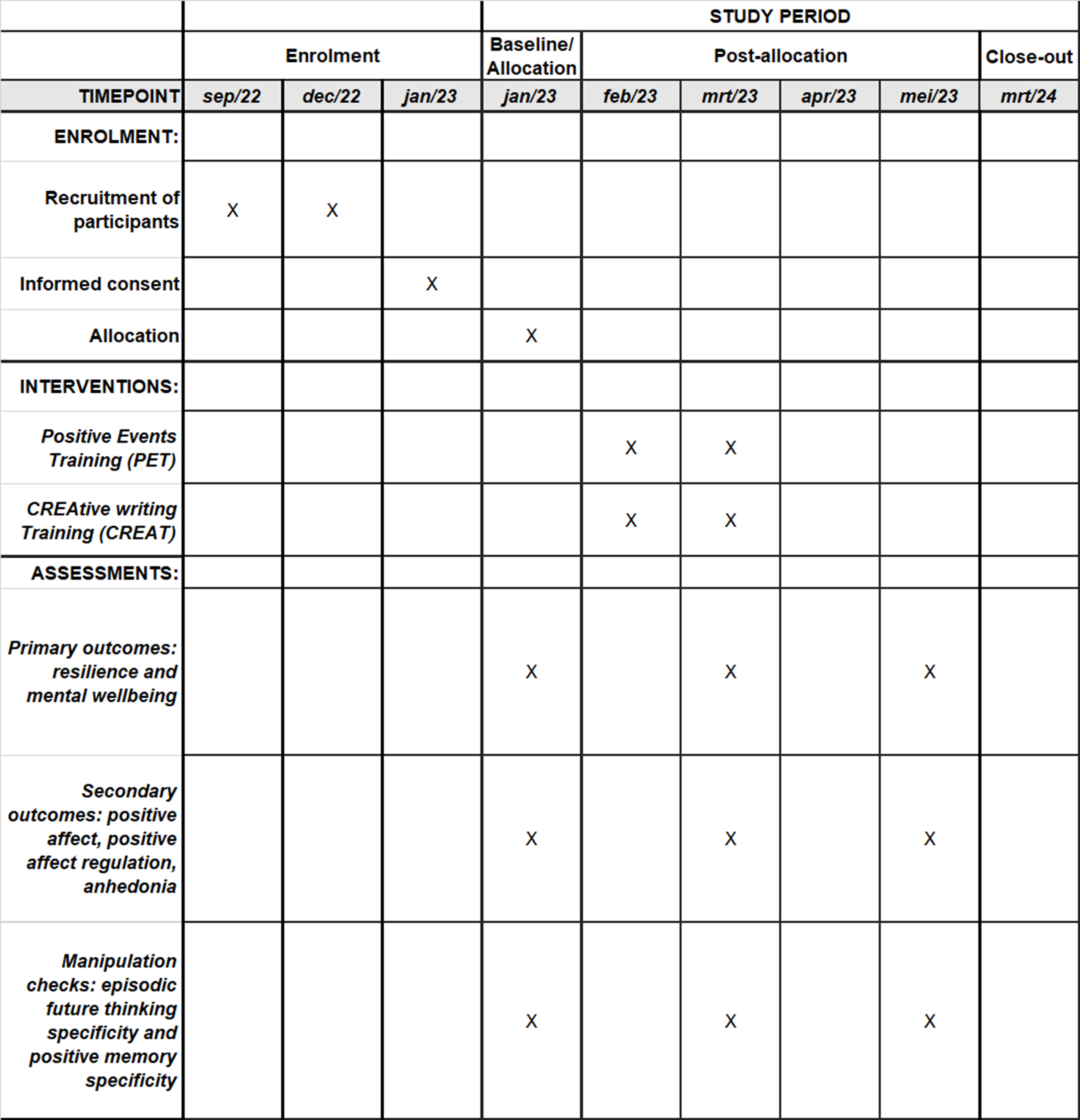

Supplement: Supplementary Figure 1 — SPIRIT schedule of enrollment, interventions, and assessments. [file Image_1.tiff]
